# Prevalent Obstacles and Predictors for People Living with Type 2 Diabetes

**DOI:** 10.1155/2012/842912

**Published:** 2012-12-02

**Authors:** L. Pilv, A. Rätsep, M. Oona, R. Kalda

**Affiliations:** Department of Policlinic and Family Medicine, University of Tartu, L. Puusepa 1a, 50406 Tartu, Estonia

## Abstract

*Background*. Type 2 diabetes (T2DM) is a chronic, progressive disease with serious micro- and macrovascular complications. A person affected by T2DM should learn to accept the new restricted lifestyle. *Aims*. The aim of the study was to identify the prevalence of obstacles in coping with daily life for people with T2DM and the magnitude of the relationships of the obstacles with various patient characteristics. *Methods*. Participants were recruited from randomly selected GPs' lists in Estonia. Respondents completed the Estonian version of the Diabetes Obstacles Questionnaire (DOQ). The statements were assessed on a 5-point scale. Biomedical and clinical variables were measured. The central tendency statistics and skewness and kurtosis for all statements were computed to find out those that reflect obstacles. Then obstacles of the DOQ were stratified. Multinomial logistic regression (MLR) was computed to estimate the influences of descriptive variables on the statements. *Results and Conclusions*. Altogether, 138 diabetic patients were enrolled in the study. Fourteen statements were identified as obstacles. Variables such as age, type of diabetes treatment, and BMI had significant effects on five of them. Younger age, BMI, and insulin nonuse were revealed as the strongest predictive characteristics for perceiving obstacles more often in coping with daily life.

## 1. Introduction

Type 2 diabetes (T2DM) is a chronic, progressive disease with serious micro- and macrovascular complications. Diabetes has a twofold additional risk, independent of other conventional risk factors, of developing a life-threatening vascular disease [1, 2]. It has been proved that effective diabetes control can prevent or delay progression of complications and reduce the death rate of T2DM [3]. However, although the latest guidelines for managing T2DM offer the best advice, treatment results are continuously poor. According to many authors, the main reason is the low adherence of patients to medical recommendations and, consequently, failure to achieve the therapeutic goals of their treatment [4–8]. During several past decades, a growing body of literature has been devoted to the investigation of the reasons for nonadherence. Numerous studies have attempted to analyze the relationships between adherence to medical advice and demographic, psychological, social, cognitive, and cultural factors between the healthcare provider and the medical system and between the disease and its treatment [9–11].

Although it is known that adherence to treatment is closely linked to T2DM patients' ability to cope with daily life, not enough attention has been given to this complex area. In comparison, the devotion to the study of relationships between physician and a patient is considerably higher [12]. It has been demonstrated that patients have many fears and they are afraid to follow all treatments recommended as necessarily in their best interests [13, 14]. Previous research has identified and described some self-perceptions and disease impacts on the daily life of patients [15–17]. However, these studies have not identified which of the patients' characteristics may have an effect on the burden in everyday life. UK and Belgian studies which used the DOQ validated the questionnaire and showed a correlation between subscales relating to management of T2DM and HbA1c, but were not intended to detect predictors for obstacles [18, 19].

On the other hand, healthcare providers, too, have difficulties in advising people with diabetes [20, 21].

According to Vermeire et al. (2001), “if there is a desire to improve adherence, then research should primarily be based on a closer understanding of patients' experience of their illness and medication rather than on the perceptions and expectations of healthcare professionals [13].” It is of the utmost importance of attaining a greater clarity of the most severe disease-related obstacles and of the factors influencing it.

We aimed to identify the prevalence of obstacles in coping with daily life for persons with T2MD from their perspective. That mainly involved finding out the possible predictors and patients' characteristics which may have an effect on these burdens in everyday life. In doing so, this would enable healthcare professionals to understand the patients' needs and fears and to provide helpful guidance.

## 2. Research Design and Methods

A questionnaire survey to research burdens and obstacles in living with T2DM was conducted in May to November 2009 in Estonia. Patient assessments were measured using the Diabetes Obstacles Questionnaire (DOQ).

The DOQ was developed to measure quantitatively the occurrence and prevalence of obstacles in coping with everyday life for diabetes patients. It was based on a comprehensive study of the literature [22] and the results of the metaethnology study EUROBSTACLE. EUROBSTACLE—an international aggregated qualitative research—was carried out in seven different European countries [23]. The results have been described elsewhere [24].

The DOQ comprises 78 statements related to eight themes (see the appendix). The statements express impacts on the everyday life of T2DM patients, such as course of diabetes (diagnosis, medical treatment, self-monitoring, and lifestyle changes), person and context, knowledge and beliefs, and relationships with healthcare provider. The Estonian version of the questionnaire was validated through translating the English version into Estonian and back into English.

The participants of this study were recruited from the GPs' lists in Estonia. Here, 741 GPs are members of the Association of Family Doctors, which makes up approximately 77% of all GPs and is the largest database of GPs in Estonia.

Eighty GPs were randomly selected from the list of the Estonian Association of Family Doctors and invited by e-mail to participate in the study. Forty-six of them (response rate 58%) agreed to recruit patients with a T2DM diagnosis, involving all types of diabetes treatment. The researchers from six participating countries (Belgium, Estonia, France, Serbia, Slovenia, and Turkey) decided on the study design of three to five consecutive patients. At the same time a similar study was carried out in all of these countries which allows a comparison of the data across the participating countries. GPs in Estonia recruited five consecutive eligible T2DM patients during their routine visit. They received a study kit including an information leaflet, an Estonian version of the DOQ, and an extract from their records with their latest clinical results, obtained no later than the previous year, and data about complications. A total of 230 questionnaires were sent to the GPs; 149 patients completed and returned the questionnaires by mail to the researchers (response rate 64.8%). The GPs and patients reasons for declining to participate were not studied in detail. Eleven patient responses were excluded from analysis because the GPs had sent extracts from their records under a separate cover. As the completed DOQs were anonymous, it was impossible to match them to the clinical data provided by the GPs. The final number of responses was 138.

Descriptive characteristics concerning age, sex, weight, height, BMI, smoking status, and disease-related variables such as glycaemic control (HbA1c), total cholesterol (Chol), low-density cholesterol (LDL-c), high-density cholesterol (HDL-c), blood pressure (BP), type of diabetic treatment, and occurrence of complications such as microangiopathy, macroangiopathy, “diabetic foot,” nephropathy, and neuropathy were studied. The frequency and percentages for categorical variables and mean, standard error of the mean, standard deviation, minimum and maximum for all quantitative variables were then computed.

The statements were assessed on a 5-point scale, graded in the questionnaire as 5 = “Strongly Agree,” 4 = “Agree,” 3 = “Neutral,” 2 = “Disagree,” and 1 = “Strongly Disagree.” The central tendency statistics and skewness and kurtosis for all statements were computed. We wanted to find out the statements that were agreed by a higher proportion of the respondents as burdens in everyday life concerning their disease. We picked the statements that corresponded to the following three criteria and defined these for “obstacles”: mode “Agree”, mean higher than 3.0, and negative skewness. Several obstacles did not show any natural ordering distribution to the values. By using multinomial logistic regression (MLR) we intended to check if any of the patients' characteristics could predict burdens. In this MLR, only the defined obstacles were used as dependent variables. Since no a priori hypothesis had been made to determine the order of entry of independent variables, all descriptive characteristics except “smoking status” were included in the analysis as independent variables. “Smoking status” encompassed 15 (10.9%) missing values.

Stratification of the dependent variable for MLR was made according to the given answers: 2 = “Strongly Agree” and “Agree” with the obstacle, 1 = “Neutral,” and 0 = “Disagree” and “Strongly Disagree” with the obstacle. We made “0” a reference category, and all parameters in the model were interpreted in reference to it. We used a backward stepwise elimination model. Parameter coefficients, asymptotic covariance and correlation matrices, likelihood-ratio tests for model and partial effects, Pearson and deviance chi-square goodness of fit, Cox and Snell, Nagelkerke, and McFadden *R* ^2^, and classification tables were all studied. The odds ratios with 95% confidence interval for variables predictors that significantly contribute to the model were computed. Means with SD for covariates and frequencies for categorical parameters were calculated to clarify whether a predictor decreased or increased the likelihood of that response category with respect to the reference category. Statistical results were considered significant at *P* < 0.05.

All quantitative data were analyzed using the IBM SPSS Statistics Version 20.

## 3. Results

In the study group of 138 participants, the mean age was 66.7 (±9.8) years and the mean duration of T2DM was 8.6 (±5.1) years. Of the participants, 45% were male. The descriptive characteristics of whole sample are presented in Table 1.

Nephropathy and macrovascular complications appeared most frequently.

Latest clinical results were HbA1c 7.1 (±1.2); Chol 5.2 mmol (±1.1) but showed a considerable range. The clinical results of the sample are presented in Table 2.

### 3.1. Prevalent Obstacles

The statistically significant majority of the respondents did not recognize most statements of the DOQ as obstacles (*N* = 54 out of 78). Only a few items (*N* = 14) were consistent with the defined obstacles' criteria. The results of the 14 obstacles are listed in Table 3.

All above-mentioned obstacles were included in MLR backward stepwise elimination analyses to find out whether the obstacles are explained by any patients' characteristics. It revealed that eight of the fourteen obstacles could be explained by some of the predictors.

### 3.2. Predictive Characteristics

Regression analysis revealed that eight of the fourteen obstacles could be explained by some of the predictors. We constructed models that only included predictors that significantly contributed to the model.

Model fitting information had been followed. Likelihood ratio tests for all models showed a significance level less than 0.05; Pseudo *R* ^2^ (Cox and Snell, Nagelkerke, and McFadden) revealed that 0.10–0.40 of the variation was explained. Classification tables showed that 53.2%–77.2% of cases were correctly classified in predicted response categories. All relevant models are summarized in Table 4.

## 4. Discussion

### 4.1. Summary of the Main Findings

The current questionnaire survey was carried out in general practices and outpatient clinics across Estonia. The clinical outcomes of the present study were comparable to that of several other studies [25–28]. The percentage of participants met the target according to ADA guidelines in 27% and 59% [29, 30] of cases. The respondents on insulin therapy were younger, showed significantly higher values in HbA1c and BMI, and their disease had lasted for a longer time in comparison with the rest of the sample.

We used the Diabetes Obstacles Questionnaire to find out what obstacles they felt in coping with daily life from the perspective of patients with T2DM. Most of the respondents judged 14 statements out of 78 as being obstacles and were indifferent about the other statements. The obstacles concerned patients' experiences of frustration and fatigue, shortage of knowledge, fear of difficulties with using insulin, and lifestyle changes, much like those already published [15, 31]. It is interesting that three of the obstacles dealt with reasons why patients could not manage physical exercises. These were no money, no time, and no enjoyment. The reason for being not delighted in exercising seems to be consistent with other research [32]. However, 69.6% of the respondents underwent difficulties with exhaustion at almost the same rate (68.8%) as those who were considered being able to change their lifestyle.

The main interest of this study was to find predictive characteristics for obstacles (Table 4). Earlier studies had made it evident that gender and clinical indicators (BMI, HDL-c, LDL-c, BP) were connected with barriers about living with diabetes [15, 33]. In the current study, eight out of fourteen obstacles had been related to some predictive characteristic. MLR analysis revealed that age, duration of the disease, type of diabetes treatment, and BMI could be predictors for consciousness of a burden. Surprisingly, the most frequent prediction was younger age. Younger respondents recognized deeper frustration (OR = 0.95, 95% CI: 0.91–0.99, *P* = 0.02), uncertainty of consequences (OR = 0.91, 95% CI: 0.87–0.96, *P* = 0.00), acknowledged shortage of sufficient understanding about their disease (OR = 0.96, 95% CI: 0.92–1.00, *P* = 0.03), doubts about insulin injections (OR = 0.94, 95% CI: 0.89–0.99, *P* = 0.02), and fear of complications (OR = 0.93, 95% CI: 0.88–0.98, *P* = 0.00). Patients nearing retirement frequently perceived some burden connected with the disease. Were they, in addition to the chronic disease, overloaded with familial or work stresses?

The patients who had been ill for a longer time acknowledged that they were not capable to fit exercise into their lifestyle (OR = 1.12, 95% CI: 1.02–1.22, *P* = 0.01). The fear of orally treated patients for switching to insulin injections was reported in earlier studies focusing exclusively on this theme [21]. Insulin emerged as a predictor in only two of the fourteen statements, focused on anxiety (OR = 0.14, 95% CI: 0.05–0.41, *P* = 0.00) and disbelief of insulin therapy (OR = 0.20, 95% CI: 0.06–0.63, *P* = 0.01). The respondents who used insulin did not confirm this fear, even if they suffered from diabetic complications (neuropathy, microangiopathy, diabetic foot syndrome). According to the statement that compared T1DM with T2DM, insulin and BMI were in covariance. Insulin users and nonusers experienced all other twelve obstacles similarly. Gender as a predicting factor came out only once. Women had been more critical over their knowledge about treatment (OR = 0.26, 95% CI: 0.10–0.65, *P* = 0.00). As we have seen, there are many obstacles about unfamiliarity with the peculiarities of the disease. The reasons for inadequate knowledge are diverse. It might take longer to get accustomed to the diagnosis and treatment options. There might have been a gap in the explanation of different treatment options from the very beginning of the disease, or else communication with other diabetes patients and diabetes associations may have been poor. The more overweight (OR = 1.29, 95% CI: 0.14–1.45, *P* = 0.00) a patient was, the bigger the problem was for him/her to watch his/her weight. They also had a higher diastolic BP and used diabetic medication infrequently. Diastolic BP was related to the statement referring to an inactive lifestyle (OR = 0.95, 95% CI: 0.91–0.99, *P* = 0.02), as well. No relationship was found between HbA1c and any studied obstacle.

### 4.2. Implications for Clinical Practice and Future Research

The current study elucidated that many obstacles are to some degree connected with the knowledge of the disease. Younger patients, especially, had many burdens. In any case, the GP's role seemed to be of the highest importance in noticing a patient's considerations, fears, and frustration arising from the disease and explaining all the aspects of the disease from the very beginning of the diagnosis. It must also be kept in mind that if there is a need to intensify treatment with insulin, then there is no reason for fear. Patients who receive insulin have found this treatment uncomplicated and easy.

Most of the participants believed that they would be able to change their lifestyle according to the advice provided by healthcare professionals. It requires a deep understanding, wisdom, and good consulting skills from the GP to encourage the capacity for building up self-efficacy and supporting a motivation for self-management [16, 34].

In future, it would be fascinating to carry out a comparable analysis using data from other countries. In addition, the sources of knowledge available to patients and the quality of information about the disease deserve further analysis.

### 4.3. Limitations of the Study

Methodological limitations to the study have to be acknowledged. Firstly, the findings presented here cannot be extrapolated to all patients with T2DM; they only allow making conclusions about those T2DM patients who visit their GP. Secondly, the study parameters involved only a limited number of variables. The obstacles identified could also have had associations with social indicators, such as educational level, marital status, and income.

## 5. Conclusions

Patients with diabetes have several burdens in everyday life. The patients picked up 14 statements that reflected their difficulties, eight of which had predictive factors. The prevalent obstacles from the patients' standpoint were connected with frustration, uncertainty and fear of difficulties with insulin, and lifestyle changes. Younger age predicted five obstacles, and insulin nonuse and BMI two obstacles.

## Figures and Tables

**Table 1:** Baseline statistics of study.

	*N* (%)
Total	138 (100)
Gender (male)	59 (45.0)
Insulin users	38 (29.0)
Tablet users	117 (90.0)
Current smokers	21 (17.9)
Macrovascular complications	21 (16.0)
Diabetic nephropathy	29 (22.1)
Microvascular complications	38 (29)
Diabetic foot	20 (15.3)
Diabetic neuropathy	35 (26.7)

**Table 2:** Clinical outcomes of the participants.

	Mean	Std. error of mean	Median	Std. deviation	Minimum	Maximum
	valid					
Age	66.7	.8	67.6	9.8	34	88
Body mass index	32.5	.5	31.6	6.0	19	50
Diabetes duration	8.6	.4	7.8	5.1	0.2	26
HbA1c	7.1	.1	6.9	1.2	5	11
Cholesterol	5.2	.1	5.2	1.1	3	10
HDL cholesterol	1.2	.0	1.1	.4	1	3
LDL cholesterol	3.4	.1	3.3	1.1	1	8
Systolic blood pressure	138.8	1.0	138.4	11.5	110	175
Diastolic blood pressure	82.7	.7	80.9	8.5	60	110

**Table 3:** The central tendency statistics and distribution parameters of the perceived obstacles.

	Mean	SE of mean	Median	Mode	SD	Skewness	Kurtosis
Using insulin makes life too complicated	3.3	.10	3	4	1.15	−.02	−1.20
I do not know as much as I need to know about the consequences of having diabetes	3.1	.10	3	4	1.19	−.02	−1.21
I do not know enough about the treatment for diabetes	3.3	.09	4	4	1.08	−.31	−.93
I believe type 2 diabetes is mild compared with type 1	3.7	.09	4	4	1.04	−.39	−.70
The way that I was told that I had diabetes made me feel confused	3.5	.10	4	4	1.21	−.22	−1.25
The way that I was told that I had diabetes made feel afraid	3.2	.10	3	4	1.17	−.10	−1.19
I am unable to fit exercise into my lifestyle	3.1	.10	3	4	1.12	−.05	−1.14
I am unable to afford the cost of exercising on a regular basis	3.1	.10	3	4	1.20	−.04	−1.06
I have not found an exercise I enjoy	3.2	.09	3	4	1.01	−.14	−.67
Weight control is real problem for me	3.5	.11	4	4	1.28	−.53	−1.03
I am able to change my lifestyle in accordance with advice from my health care professional(s)	3.6	.09	4	4	1.05	−.87	.06
Self management of diabetes is difficult to maintain because diabetes complications are not immediate	3.6	.09	4	4	1.01	−.62	−.49
Good control of diabetes involves a lot of sacrifice	3.3	.10	4	4	1.13	−.16	−1.16
I feel that I would like to take a holiday from my diabetes	3.7	.09	4	4	1.08	−.77	−.22

**Table 4:** Predictors of patients' assessments about daily obstacles in living with T2DM by multinomial logistic analysis. Odds ratios with 95% confidence interval (OR; 95% CI).

	Disagree		Neutral				Agree			
	*N* (%)	Mean (SD)	*N* (%)	Mean (SD)	OR (95% CI)	*P*	*N* (%)	Mean (SD)	OR (95% CI)	*P*
	Using insulin makes life too complicated									
	42		29				67			
Age (year)		67.8 (8.9)		69.1 (9.7)	1.02 (.96; 1.08)	0.58		65 (10.2)	.94 (.89; .99)	0.02
Use of insulin	23 (57.5)		3 (10.7)		.10 (.02; .39)	0.00	12 (19.4)		.14 (.05; .41)	0.00
Neuropathy	19 (45.2)		7 (24.1)		1.33 (.32; 5.53)	0.69	12 (18.2)		.13 (.04; .47)	0.00
Microangiopathy	17 (40.5)		4 (13.8)		.24 (.05; 1.18)	0.08	39 (28.5)		1.88 (.60; 5.82)	0.28
Nephropathy	8 (19.0)		4 (13.8)		1.09 (.24; 4.98)	0.91	18 (27.3)		4.44 (1.28; 15.44)	0.02
	I believe type 2 diabetes is mild compared with type 1									
	22		30				75			
Systolic BP		136.4 (8.6)		141.6 (10.3)	1.09 (1.02; 1.16)	0.01		138.5 (12.4)	1.06 (1.00; 1.12)	0.04
Use of insulin	12 (54.5)		12 (40)		.62 (.16; 2.35)	0.48	13 (17.1)		.20 (.06; .63)	0.01
Use of tablets	16 (72.7)		31 (96.9)		26.37 (2.46; 282.72)	0.01	74 (92.5)		6.36 (1.38; 29.24)	0.02
BMI		34.8 (5.9)		31.9 (4.6)	.87 (.78; .97)	0.05		32.0 (6.2)	.91 (.84; 1.00)	0.04
	I do not know enough about the treatment for diabetes									
	39		23				65			
Age (year)	69.0 (9.3)		69.0 (9.0)		1 (.95; 1.06)	0.97	65.0 (10.0)		.96 (.92; 1.00)	0.03
	I do not know as much as I need to know about the consequences of having diabetes									
	53		17				56			
Age (year)		69.3 (8.9)		67.9 (11.2)	.98 (.92; 1.05)	0.56		63.9 (9.5)	.91 (.87; .96)	0.00
Gender (male)	27 (48.2)		11 (64.7)		1.40 (.41; 4.79)	0.60	22 (36.7)		.26 (.10; .65)	0.00
	I am unable to fit exercise into my lifestyle									
	53		21				52			
T2DM duration (y)		8.1 (4.1)		6.8 (3.4)	.91 (.80; 1.03)	0.15		9.3 (6.1)	1.12 (1.02; 1.22)	0.01
Diastolic BP		81.2 (8.6)		82.3 (7.1)	.99 (.92; 1.06)	0.68		84.6 (8.2)	1.08 (1.02; 1.15)	0.01
	Weight control is real problem for me									
	40		4				83			
Use of tablets	42 (97.7)		4 (100)				76 (87.4)		.07 (.01; .72)	0.02
BMI		29.1 (4.6)		31.1 (4.2)	1.12 (.86; 1.45)	0.41		34.1 (5.7)	1.29 (1.14, 1.45)	0
Diastolic BP		79.8 (7.2)		85.3 (6.7)	1.10 (.96; 1.26)	0.16		84.2 (8.5)	1.09 (1.02; 1.16)	0.02
	Self-management of diabetes is difficult to maintain because diabetes complications are not immediate									
	27		16				86			
Age (year)		71.1 (9.4)		67.6 (9.4)	.94 (.88; 1.01)	0.10		65.2 (9.7)	.93 (.88; .98)	0.00
Diabetic foot	1 (3.6%)		3 (17.6)		6.91 (.65; 73.64)	0.11	16 (17.4)		7.15 (.88; 58.10)	0.07
	Good control of diabetes involves a lot of sacrifice									
	43		19				67			
Age (year)		69.0 (9.1)		68.5 (10.7)	.98 (.93; 1.04)	0.56		64.7 (9.8)	.95 (.91; .99)	0.02

The “agree” group (category = 2) was the largest group and the smallest was the “neutral” group. The predictive characteristics showed a significant effect on the “agree” group but seldom on the “neutral” group respondents. “Age” was significant in five obstacles by agreed participants but on no occasion by neutrals.

## Appendix

The Diabetes Obstacles Questionnaire (DOQ). Scale 1—Medication Obstacles Scale.

1. I do not feel I am being prescribed the medication that is right for me
2. I do not feel I am being prescribed the medication dose that is right for me
3. I do not know what to do about taking my medication when I am feeling unwell
4. Taking insulin makes life too complicated
5. Taking insulin means my diabetes is getting worse
6. People treat insulin users differently
7. I am not in a convenient place when it is time to take my medication
8. I often forget to take my medication
9. My medication causes unwanted side effects
10. I feel resentful that I have to take my medication.

Scale 2—Self-Monitoring Obstacles Scale.

1. I find it especially hard to test when I'm busy
2. Self-monitoring makes me feel frustrated
3. Self-monitoring makes me fearful of a high reading
4. I do not feel that self-monitoring is helping me to control my diabetes
5. I find it too uncomfortable to self-monitor.

Scale 3—Obstacles of Knowledge and Beliefs Scale.

1. I do not know as much as I need to know to manage my diabetes
2. I have difficulty accessing information that is relevant to me personally
3. I have difficulty understanding the information from literature
4. I have difficulty understanding the information from healthcare professionals
5. I think that the information on diabetes is not consistent
6. I do not know as much as I need to know about the consequences of having diabetes
7. I do not know enough about the treatment for diabetes
8. I believe Type 2 diabetes is mild compared with Type 1
9. I do not know enough about the benefits of diabetes treatment for me personally
10. I do not believe the consequences of Type 2 diabetes are serious.

Scale 4—Obstacles of Diagnosis Scale.

1. The way that I was told that I had diabetes made me feel confused
2. The way that I was told that I had diabetes made me feel afraid
3. The way that I was told that I had diabetes made me feel that it was not a serious condition
4. The was that I was told that I had diabetes did not motivate me to manage my diabetes well
5. I was not given as much information as I needed about the consequences of having diabetes
6. The way that I was told that I had diabetes made me feel guilty.

Scale 5—Obstacles of Relationships with the Healthcare Professionals Scale.

1. I feel my questions about diabetes are not answered
2. I feel I am not listened to
3. I feel my judgment is not trusted in managing my diabetes
4. I am not advised at all on what to do about my diabetes
5. I am not assisted in setting realistic targets for changing my lifestyle
6. Treatment alternatives are not explained to me
7. I have not been told what to expect from my diabetes
8. I have not been told what to expect from my treatment
9. I do not feel I am part of the diabetes team
10. The good and bad aspects of my choice have not been discussed with me
11. I am not asked at all which choice I would prefer
12. Talking about my diabetes with members of the diabetes team does not make me feel better
13. Adjustments to my diabetes plan cannot be discussed
14. I feel threatened when I go for a checkup
15. I feel a sense of powerlessness when consulting with nurses
16. I feel a sense of powerlessness when consulting with doctors
17. Clinic times are inconvenient for me
18. I have to spend too much time waiting in clinics.

Scale 6—Obstacles of the Lifestyle Changes Scale.

1. My diabetic diet spoils my social life
2. I generally still feel hungry after finishing a meal
3. My diabetes has placed a strain on my personal relationships
4. There is little hope of leading a normal life when you have diabetes
5. Changes in my diet have put a strain on my family
6. I have difficulty sticking to my diet when I am away from home
7. I feel resentful that I am obliged to change my eating habits
8. I am unable to fit exercise into my lifestyle
9. I am unable to afford the cost of exercising on a regular basis
10. I haven't found an exercise I enjoy
11. I lack the motivation to exercise
12. Weight control is a real problem for me
13. I am able to change my lifestyle in accordance with advice from healthcare professional(s).

Scale 7—Obstacles of Coping with Diabetes Scale.

1. Self-management of diabetes is difficult to maintain because diabetes complications are not immediate
2. Good control of diabetes involves a lot of sacrifice
3. I find it difficult to get into a suitable routine to cope with my treatment plan
4. I am not convinced that the treatment I receive for my diabetes is effective
5. I feel overwhelmed by the responsibility of having to take my medication
6. I feel that I would like to take a holiday from my diabetes
7. I eat something I should not rather than say I have diabetes
8. I feel that my family would like to take a holiday from my diabetes.

Scale 8—Obstacles of the Advice and Support Scale.

1. I am not convinced healthcare professionals believe the treatment I receive for my diabetes will work
2. I am told too often what I should and should not be doing to manage my diabetes
3. Constantly repeating what I should be doing to manage my diabetes makes me do it less
4. I am criticized too often about the way I manage my diabetes
5. I would manage my diabetes much better if I had more encouragement socially
6. I feel very alone with my diabetes
7. I feel I get little support from my family
8. I feel I get little support from my friends.
